# Fitting of TC model according to key parameters affecting Parkinson's state based on improved particle swarm optimization algorithm

**DOI:** 10.1038/s41598-022-18267-9

**Published:** 2022-08-17

**Authors:** Chunhua Yuan, Xiangyu Li

**Affiliations:** School of Automation and Electrical Engineering, Shenyang Ligong University, Shenyang, 110159 China

**Keywords:** Neuroscience, Neurology

## Abstract

Biophysical models contain a large number of parameters, while the spiking characteristics of neurons are related to a few key parameters. For thalamic neurons, relay reliability is an important characteristic that affects Parkinson's state. This paper proposes a method to fit key parameters of the model based on the spiking characteristics of neurons, and improves the traditional particle swarm optimization algorithm. That is, a nonlinear concave function and a Logistic chaotic mapping are combined to adjust the inertia weight of particles to avoid the particle falling into a local optimum in the search process or appearing premature convergence. In this paper, three parameters that play an important role in Parkinson's state of the thalamic cell model are selected and fitted by the improved particle swarm optimization algorithm. Using the fitted parameters to reconstruct the neuron model can predict the spiking trajectories well, which verifies the effectiveness of the fitting method. By comparing the fitting results with other particle swarm optimization algorithms, it is shown that the proposed particle swarm optimization algorithm can better avoid local optima and converge to the optimal values quickly.

## Introduction

In recent years, neurological diseases caused by abnormal brain function have become a class of diseases that seriously affect human physical and mental health^1^. However, due to the limitations of human experiments, model-based research is more common^2^. The researches on neurological diseases have given birth to the demand for neuron calculation and modeling^3^. Generally speaking, computational models can verify the proposed hypotheses, reflect individual differences through the internal structure and parameters of the model, and link the basic neural circuits with brain functions, thereby providing new tools for understanding neurological diseases. Since the specific characteristics of the model depend on the neuron, it is necessary to fit the computational model to the electrophysiological record^4^.

The conductance-based biophysical model can predict various spiking patterns with high accuracy, but the large network simulation of this type of model is computationally expensive^5^. Therefore, scientists have proposed various simple phenomenological spiking models as an alternative, such as linear integrate-and-fire model and various extensions to it—square integrate-and-fire model^6^, exponential integrate-and-fire model^7^, and Poisson generalized integrate-and-fire model^8^, etc. In recent years, most of the fitting and prediction based on neuron models have adopted simple spiking models rather than detailed conductance models describing cell biophysics. Hertäg used standard f-I curves obtained by in vitro electrophysiologists to fit the adaptive integrate-and-fire neuron model and the adaptive exponential integrate-and-fire model^9^. Ghosh et al. implemented an adaptive exponential integrate-and-fire model on the NEURON simulation platform, estimated its parameters to describe the reference neuron, and verified the initiation dynamics of the model^4^. Pozzorini proposed a protocol combined with automatic patch clamp recording technology, which can efficiently transform a large amount of in vitro electrophysiological data into a spiking neuron model^10^. As large-scale network simulation has gradually become a hot research topic, more realistic models describing neuron characteristics are needed^11^. However, the complex structure and numerous parameters of the biophysical model reduce its computational efficiency and increase the computational difficulty. And serious overfitting is likely to occur. While in most biophysical models, only a few key ion channel parameters affect the intrinsic spiking characteristics of neurons^12^.

Parkinson's disease is a common degenerative disease of the nervous system, which is more common in the elderly. The clinical manifestations are static tremor, muscle rigidity, bradykinesia and postural instability^13^. The main pathological changes of Parkinson's disease are in the basal ganglia and thalamus neurons of the brain. When more than 70% of the substantia nigra dopaminergic neurons in the basal ganglia are destroyed, a significant decrease in striatal dopamine is caused and the normal function of the basal ganglia is hindered, inhibiting the relay function from thalamus neurons to cortex, thus leading to the symptoms of parkinson's disease^14,15^. The hindrance of the relay function of thalamic neurons is mainly due to the impact of the rebound cluster spiking behavior of the basal ganglia. Researchers have found that this rebound cluster spiking behavior is mainly related to the low threshold calcium ion current^15,16^. Therefore, the important parameters are fitted based on the significant spiking characteristics affecting Parkinson's state in this paper.

Since the signal transmission of the nervous system contains complex nonlinear processes^17^, it is difficult to obtain an effective system model using traditional linear fitting methods. Mitra et al. used the AugMAT model to predict the experimental discharge data, and adopted the simplex method and the gradient-based optimization method to fit the model parameters^18^. These algorithms are local optimization algorithms, that is, the search effect depends on the initial values of the parameters. In recent years, some evolutionary algorithms have been introduced into the parameter fitting process, such as genetic algorithm^19^, differential evolution algorithm^20^ and particle swarm optimization algorithm^21^. These algorithms optimize the optimal solution through multi-dimensional parallel work. And the search process has higher efficiency and better results than traditional algorithms. The particle swarm optimization algorithm is a multi-dimensional and large-scale intelligent optimization algorithm proposed by Kenney and Eberhart^22^. Bangyal et al. gave a comprehensive survey of the various particle swarm optimization and DE initialization approaches based on the family of quasirandom sequences such as Sobol sequence, Halton sequence, and uniform random distribution^23^. They show that the low-discrepancy sequences-based initialization performed exceptionally better than a uniform random number. Pervaiz et al. presented a thorough overview of different particle swarm optimization initialization approaches which were dependent on semi-arbitrary successions systems and the best in class in the populace instatement was uncovered^24^. It was found that the proposed version of the algorithm is more effective in finding the global minimum of unimodal and multimodal test functions. Although there are many advantages of the particle swarm optimization algorithm, particles are prone to fall into local optima during the search process, or the case of premature convergence occurs^12^. Using a single method to improve particle swarm optimization algorithm may still fall into a local optimum. Therefore, a combination of nonlinear concave function and Logistic chaotic mapping is adopted to improve the particle swarm optimization algorithm in this paper.

In summary, we propose a method to fit the key parameters in the thalamic cell (TC) model and predict spiking trajectories of the Parkinson's state and the normal state. The particle swarm optimization algorithm is improved, and the fitness function is selected based on the spiking characteristics of the thalamic neurons in the Parkinson’s state. After the key parameters that affect the Parkinson’s state are fitted, the TC neuron model is reconstructed using the fitted parameters. The spiking trajectories of the reconstructed neuron model are compared with that of the original model to verify the effectiveness of the improved algorithm. Finally, four improved particle swarm optimization algorithms are compared with to prove the advantages of the proposed algorithm.

## Results

### System description

**Figure 1:**
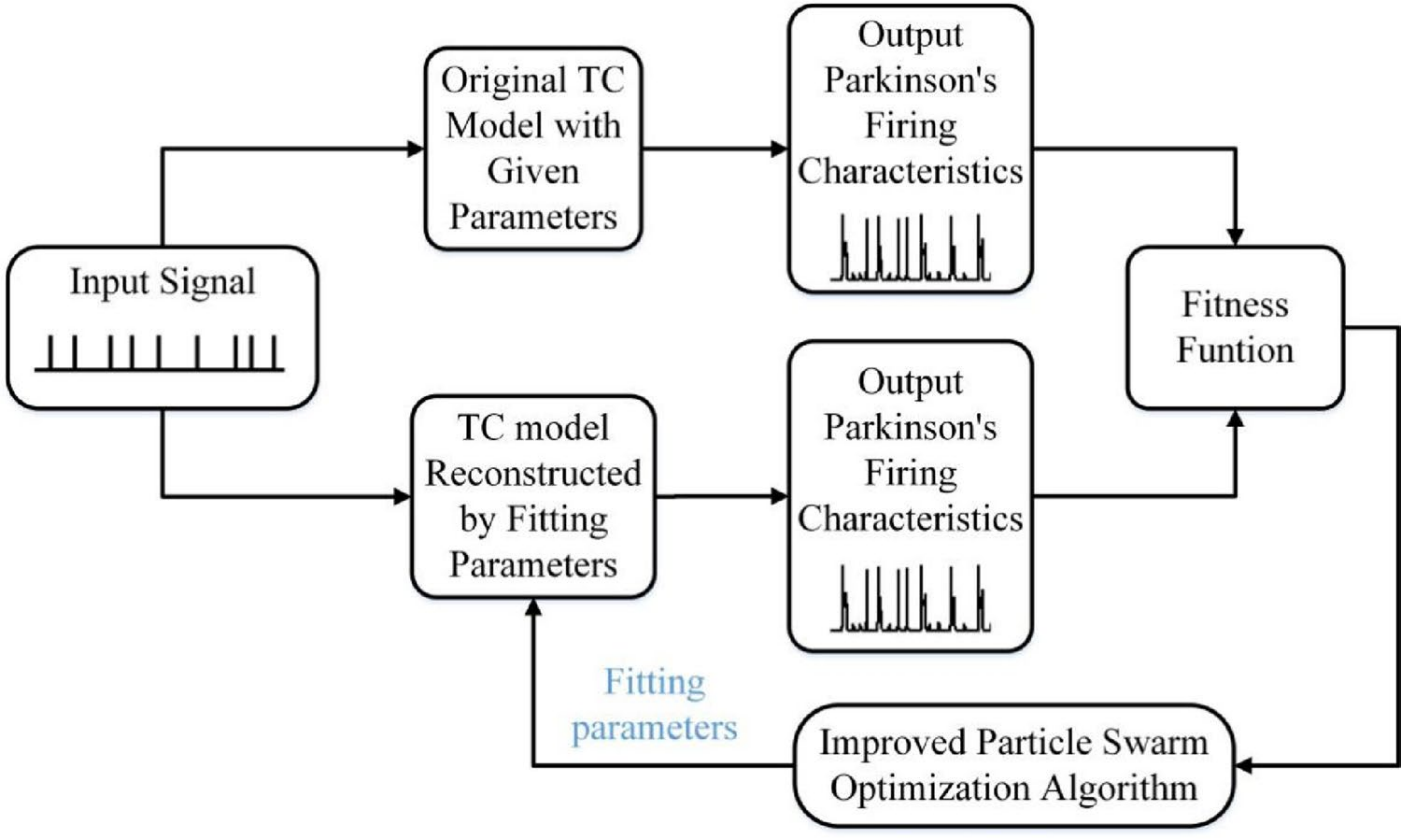
Fitting process of the TC model based on the improved particle swarm optimization algorithm.

The TC model parameter fitting process based on the improved particle swarm optimization algorithm is shown in Fig. 1. Firstly, by inputting signals to the TC model with given parameters, the output firing data is obtained as standard data. Then give the same input signals to the reconstructed model with unknown key parameters, and compare its output data with the standard output data through the selected fitness function. Finally use the improved particle swarm optimization algorithm proposed in this paper to fit the unknown key parameters.

### Fitness function of the TC model

The TC model in normal state can relay the input signal without losing the spiking information. While the TC model in Parkinson’s state cannot relay the input signal well, that is, the output signal cannot match the input signal well, resulting in the spiking information loss. Also the reliability of the relay is greatly reduced. Therefore, relay reliability is an important spiking characteristic of the TC model. In order to quantify relay reliability of the TC model, we define:

1 $$RI = 1 - error\;index$$

2 $$error\;index = \frac{missed\;spikes + bad\;spikes}{{total\;inputs}}$$

where $error\;index$ is the error rate of the relay, including the spiking information of missed relay and the spiking information with relay error. Obviously, the value range of $RI$ is between [0, 1]. 0 means that the output signal does not match the input signal at all, and 1 means that the output completely matches the input signal. Generally speaking, there is no obvious boundary between the TC model in normal state and in Parkinson's state. But it is generally believed that the TC model is in normal state if $RI > 0.9$, and it is in Parkinson's state if $RI < 0.5$.

In addition to the reliability of the relay, we also select the total number of spikes, the average value of the spike peak and the average value of the subthreshold voltage together as the spiking characteristics of the TC model for parameter fitting to ensure the uniqueness of the spiking trajectory. Therefore, the fitness function for parameter fitting of TC model is selected as follows:

3 $$\begin{aligned} q_{T} & = w_{1T} \sum {(RI_{rec} - RI_{ori} )}^{2} + w_{2T} \sum {(N_{rec} - N_{ori} )^{2} } \\ & \quad + w_{3T} \sum {(PK_{rec} - PK_{ori} )}^{2} + w_{4T} \sum {(ST_{rec} - ST_{ori} )}^{2} \\ \end{aligned}$$

where $RI_{i}$, $N_{i}$, $PK_{i}$ and $ST_{i}$ ($i$ = rec, ori) respectively represent the relay reliability, the number of spikes, the average spike peak value and the average subthreshold voltage value of the reconstructed TC model and the TC model with given parameters. $w_{1T}$, $w_{2T}$, $w_{3T}$ and $w_{4T}$ are the weight of each part respectively.

### Fitting process of the TC model

Given the input signal $I_{SM}$ of the TC model as a square wave pulse signal:

4 $$I_{SM} = A_{SM} H\left( {sin\left( {\frac{2\pi t}{{\rho_{SM} }}} \right)} \right) \times \left( {1 - H\left( {sin\left( {\frac{{2\pi (t + \delta_{SM} )}}{{\rho_{SM} }}} \right)} \right)} \right)$$

where the amplitude is *A*_SM_ = 5 pA/μm^2^, the period is *ρ*_SM_ = 25 ms, the duration is *δ*_SM_ = 5 ms, and $H$ is the Heaviside step function:

5 $$H(x) = \left\{ {\begin{array}{*{20}l} {0,} \hfill & {x < 0} \hfill \\ {1,} \hfill & {x \ge 0} \hfill \\ \end{array} } \right.$$

**Figure 2:**
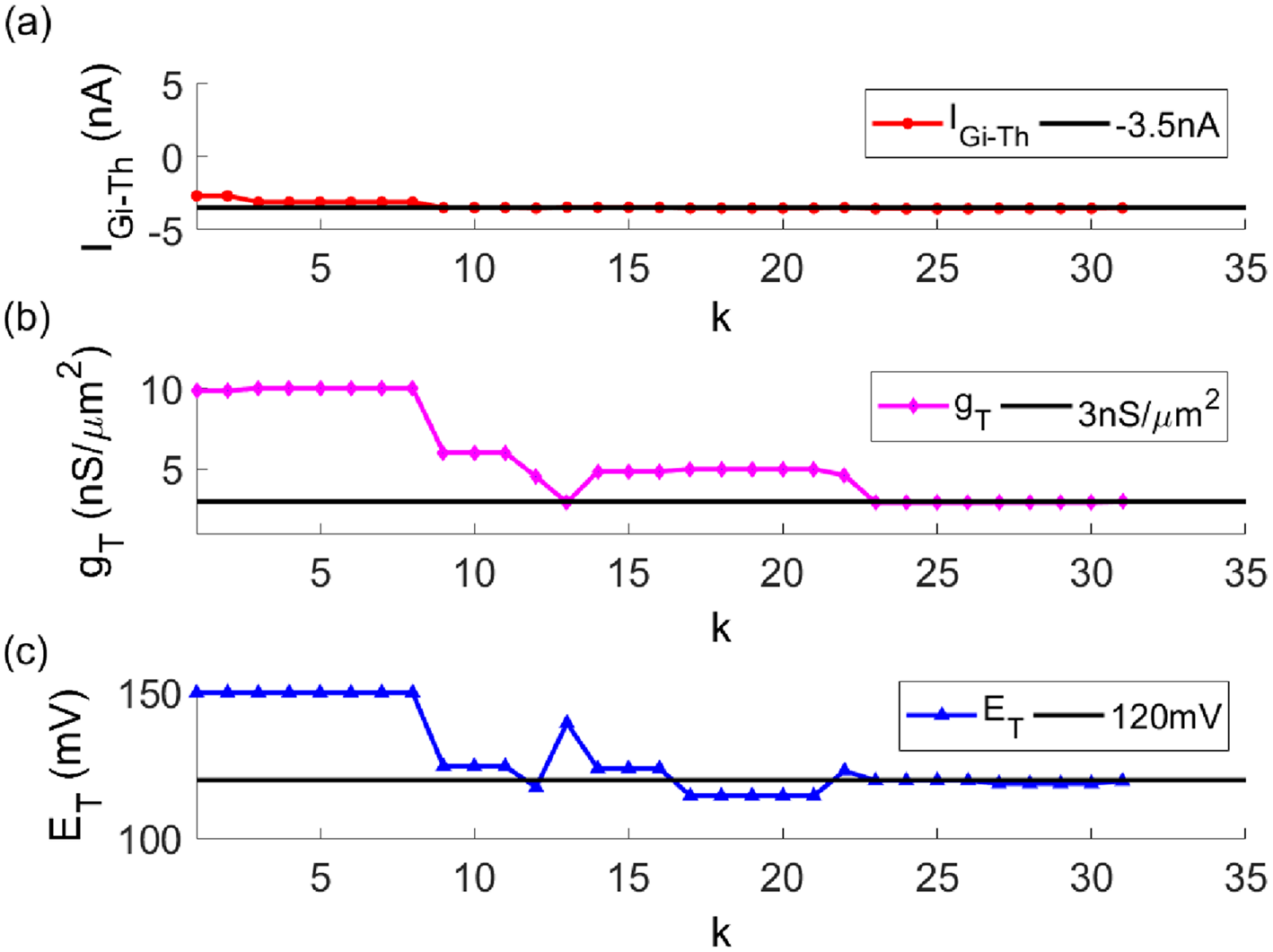
The fitting process of three key parameters of the TC model in Parkinson's state. (**a**) $I_{Gi \to Th}$ (**b**) $g_{T}$ (**c**) $E_{T}$.

**Figure 3:**
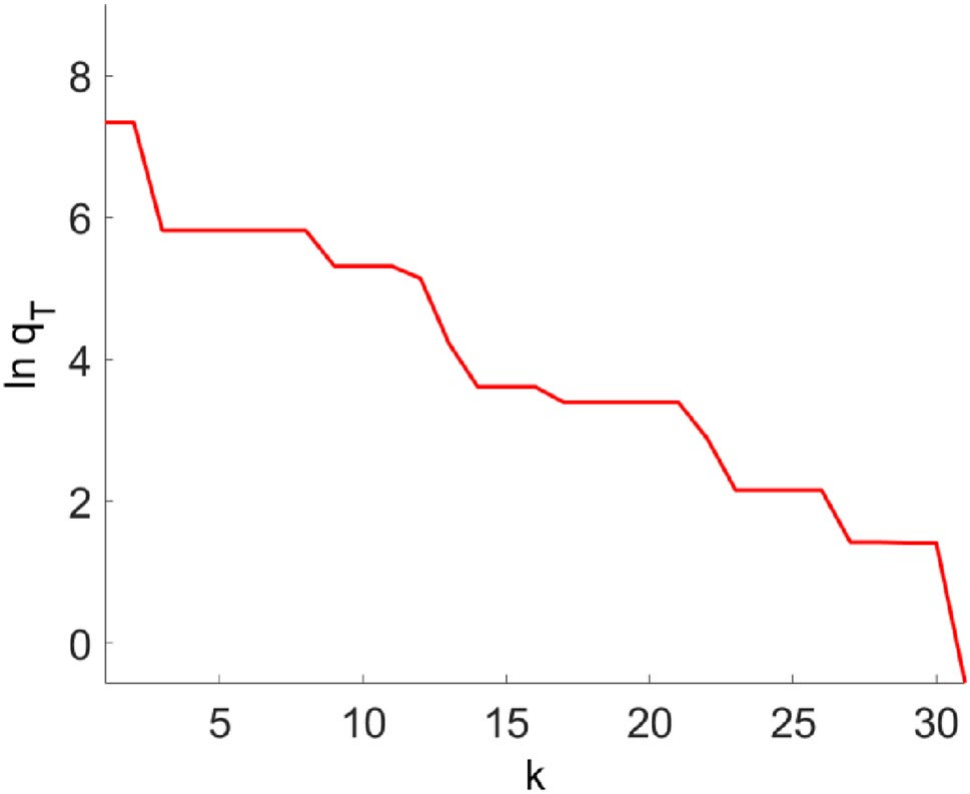
The fitness function $q_{T}$ changes with the iteration number $k$.

In the TC model with given parameters, the key parameters of Parkinson’s state are given as $[I_{Gi \to Th},g_{T},E_{T} ]$ = [− 3.5, 3, 120], which refers to the data in Ref. ^26^ and comes from biophysical experiments. In the TC model to be fitted, except for the three parameters $I_{Gi \to Th}$, $g_{T}$ and $E_{T}$, the remaining parameters are set as constants. The improved particle swarm optimization algorithm are used to fit three important parameters. The fitting process of the three parameters is shown in Fig. 2. The red, pink and blue curves represent the parameters $I_{Gi \to Th}$, $g_{T}$ and $E_{T}$ to be fitted respectively. It can be seen from the figure that as the number of iterations increases, the three parameters all tend to the given value from the initial value. After a period of oscillation, it finally stops after the 31st iteration. The change of the fitness function with the number of iterations is shown in Fig. 3. It is shown in the figure that as the number of iterations increases, the fitness function gradually converges. When the iteration termination condition is met, the iteration stops. The values of the three parameters are the final iteration results, that is $[I_{Gi \to Th},g_{T},E_{T} ]$ = [− 3.4991, 3.0215, 119.6532].

### Effects of the fitting method

**Figure 4:**
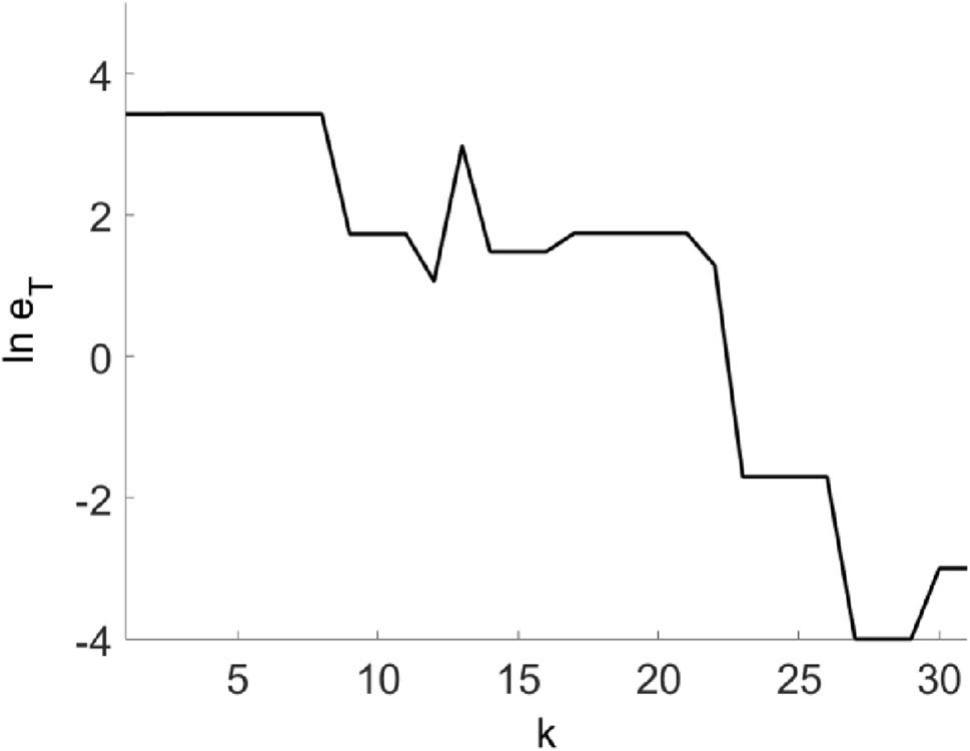
The parameter error $e_{T}$ changes with the iteration number $k$.

Define the parameter error of the TC model as:

6 $$e_{T} = e_{{I_{Gi \to Th} }}^{2} + e_{{g_{T} }}^{2} + e_{{E_{T} }}^{2}$$

where $e_{{I_{Gi \to Th} }}$, $e_{{g_{T} }}$ and $e_{{E_{T} }}$ are the errors between the optimal solution and the given value of the three parameters $I_{Gi \to Th}$, $g_{T}$ and $E_{T}$. It can be seen from Fig. 4 that as the number of iterations increases, the parameter error to be fitted gradually decreases. And the final iteration error satisfies $\ln e_{T} < - 2$.

**Figure 5:**
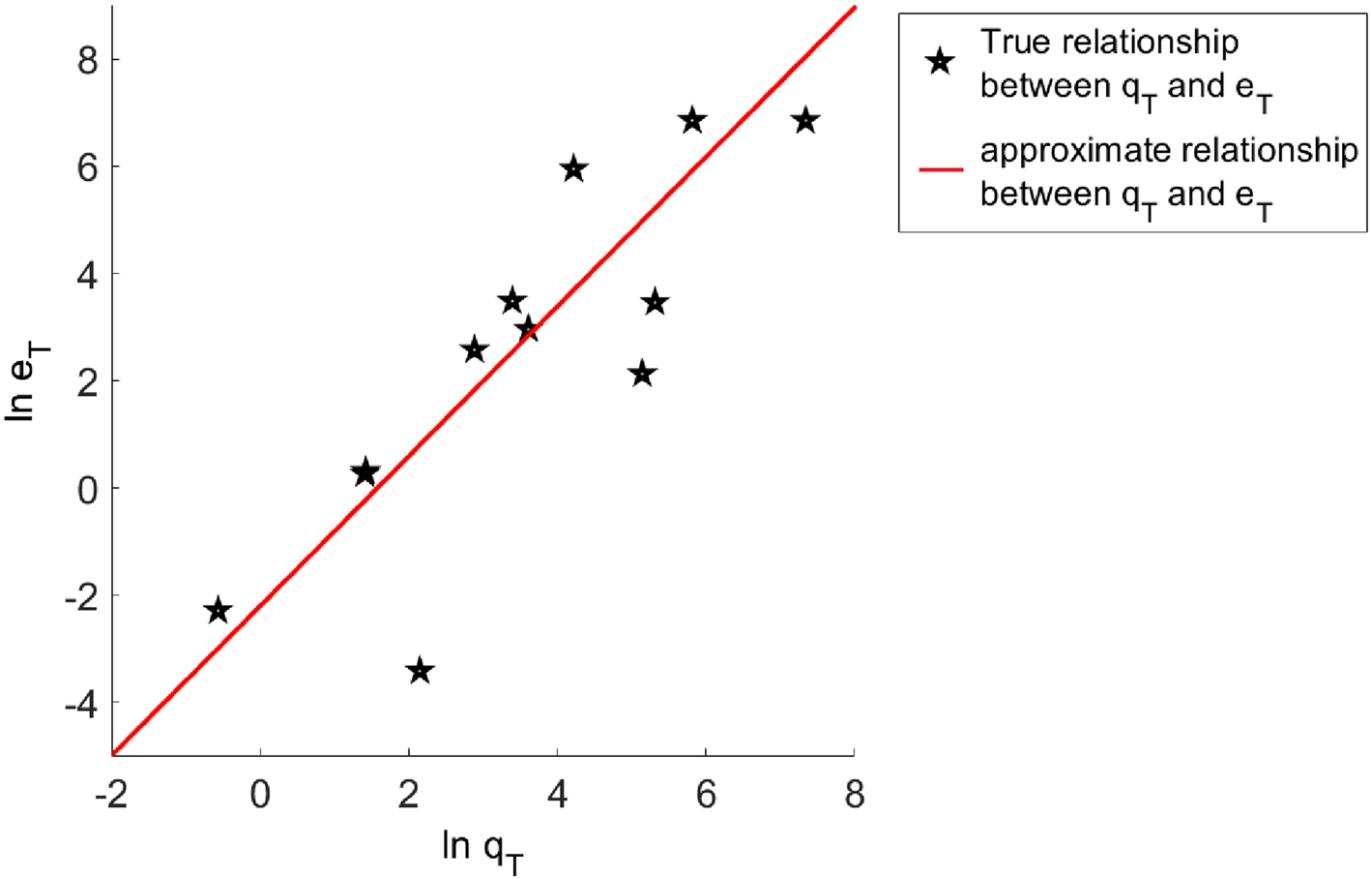
The relationship between fitness function $q_{T}$ and parameter error $e_{T}$.

In order to explore the relationship between the fitness function of the optimal particle and the parameter error, we perform multiple simulation experiments on the TC model, and statistically analyze the results. The logarithmic relationship between the fitness function $q_{T}$ and the parameter error function $e_{T}$ of multiple simulation results is shown in Fig. 5. The results show that the relationship between the two is almost linear. Therefore, it can be ensured that when the fitness function is small enough, the error between the optimal solution and the true value is small enough.

**Figure 6:**
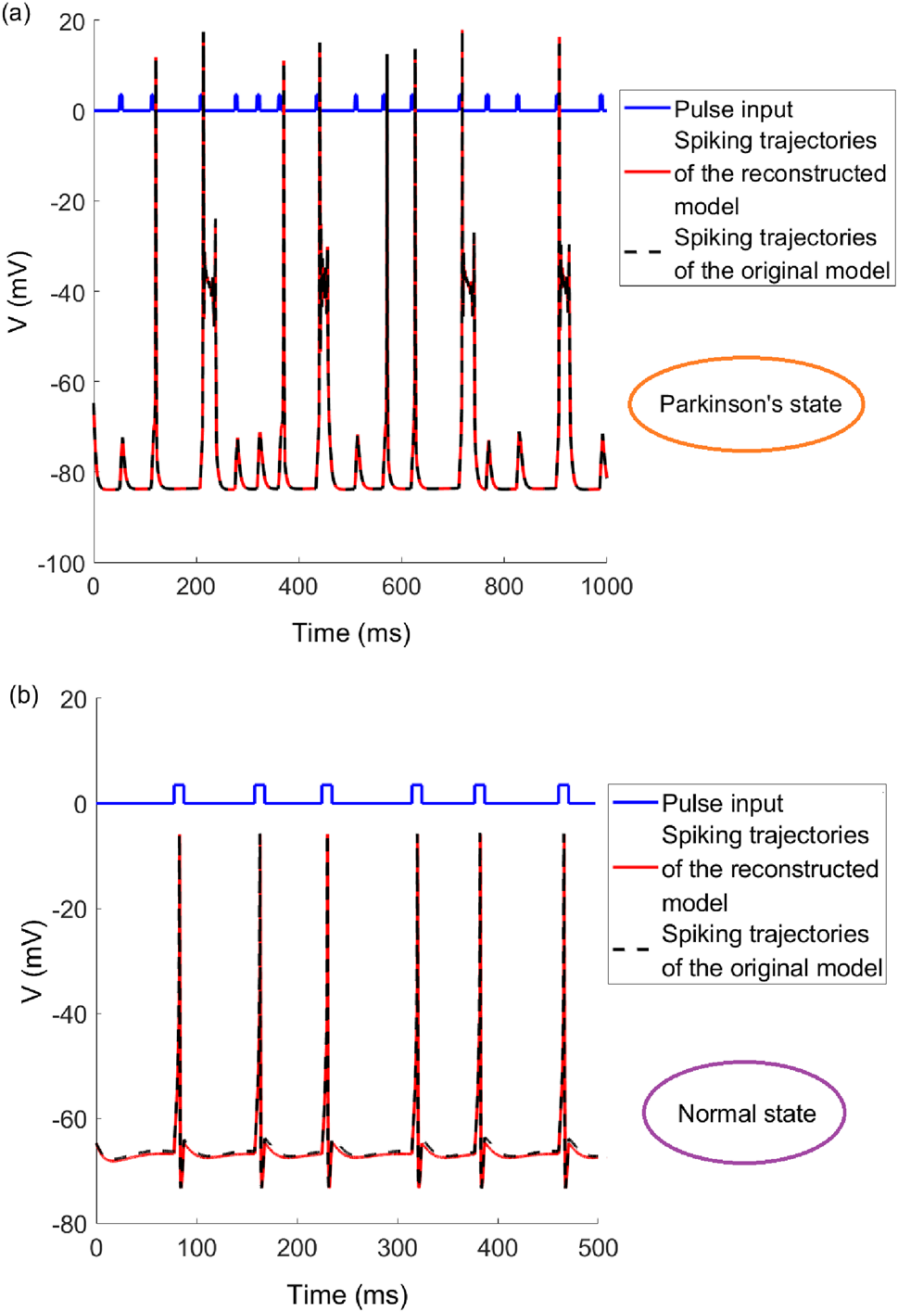
The spiking trajectories of the TC model with given parameters and the reconstructed TC model with fitted parameters in (**a**) Parkinson's state (**b**) normal state under Poisson input. The black curves represent the spiking trajectories of the original model with given parameters, and the red curves represent the spiking trajectories of the reconstructed model. The blue curves are the Poisson pulse inputs.

To test the effectiveness of the fitted parameters, we use the fitted parameters to reconstruct the TC model and check whether the spiking trajectories of the reconstructed model and the original model are consistent. Figure 6a shows the spiking trajectories of the TC model with given parameters and the reconstructed model with fitted parameters in Parkinson’s state. The black curve is the original model, the red curve is the reconstructed model, and the blue curve is the Poisson pulse input. It can be seen that the two curves overlap perfectly. Figure 6b shows the spiking trajectories of the given parameter model and the reconstructed model in normal state of the TC model. The black curve is the original model, the red curve is the reconstructed model, and the blue curve is the impulse input. The curves can also overlap well. It shows that whether it is in Parkinson's state or in normal state, the method proposed in this paper can fit the key parameters of the TC model effectively.

## Discussion

The improved particle swarm algorithm adopted in this paper is to adjust the inertia weight of particles through the combination of nonlinear concave function and Logistic chaotic mapping. In the particle swarm optimization algorithm, the inertia weight has a great influence on the convergence of the algorithm. In the classical particle swarm optimization algorithm, the inertia weight is usually chosen as a constant. While the usual improved algorithm generally selects the inertia weight as a certain function, which often causes the algorithm to fall into a local optimum and not to converge. In order to compare with the improved particle swarm optimization algorithm proposed in this paper, three key parameters of the TC model are fitted by selecting inertia weight as a constant, a single determined linear function and two sets of dynamic nonlinear concave functions, respectively.

**Figure 7:**
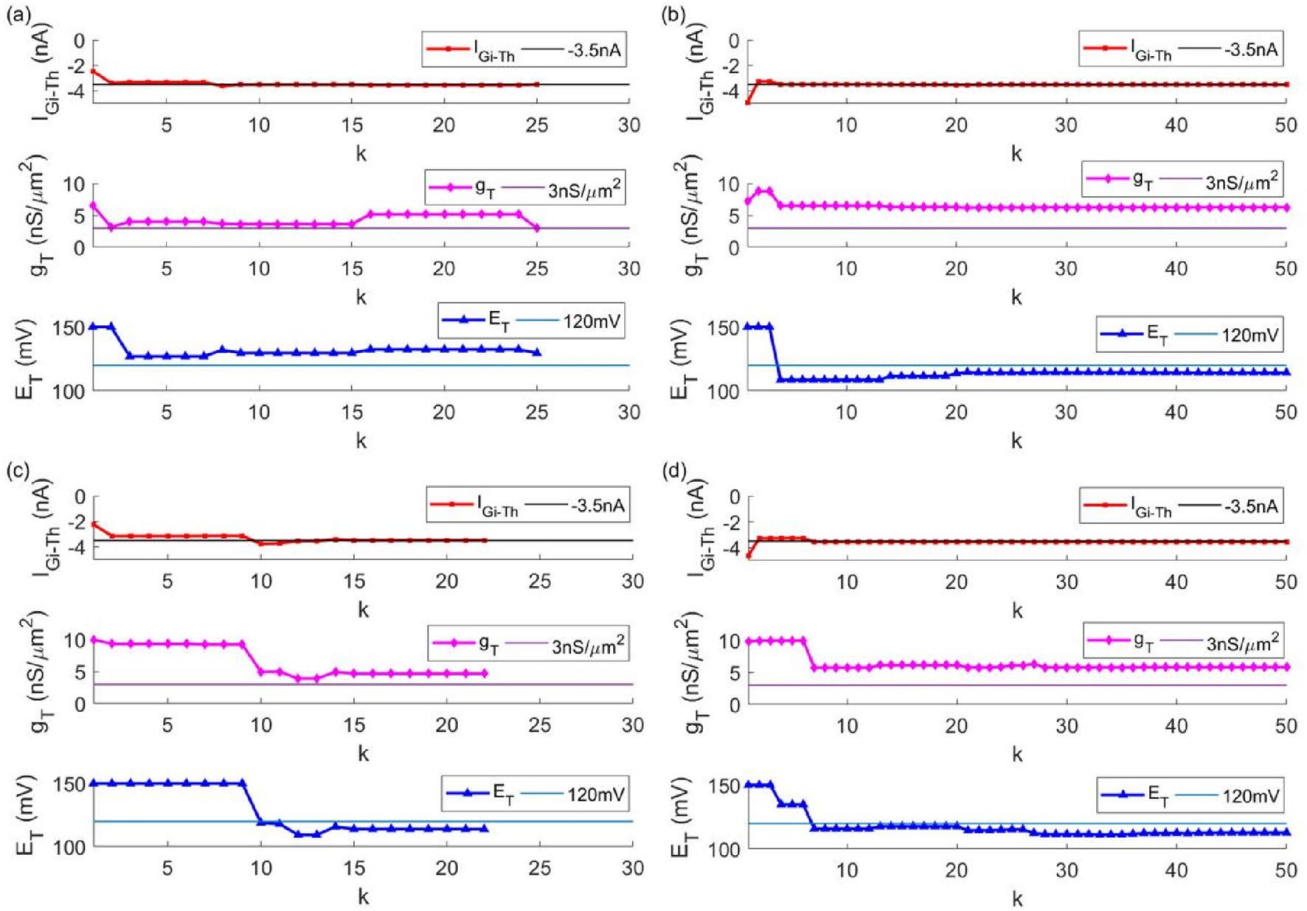
The fitting process of the three key parameters $I_{Gi \to Th}$, $g_{T}$ and $E_{T}$ of the TC model. The inertia weight is: (**a**) a constant (**b**) a determined linear function (**c**) the first single dynamic nonlinear concave function (**d**) the second single dynamic nonlinear concave function.

Figure 7 shows the fitting process of the three parameters $I_{Gi \to Th}$, $g_{T}$ and $E_{T}$ to be fitted in the TC model, in which: the inertia weight of Fig. 7a is a constant, and $\omega = 0.7$ is selected. The inertia weight of Fig. 7b adopts a determined linear function:

7 $$\omega = \omega_{\max } - (\omega_{\max } - \omega_{\min } )*\left( {\frac{k}{{k_{\max } }}} \right)$$

A single dynamic nonlinear concave function is used to improve the inertia weight in Fig. 7c, which is shown in Eq. (13) on Methods part of this paper. Figure 7d uses another nonlinear concave function to improve the inertia weight:

8 $$\omega = (\omega_{\max } - \omega_{\min } )*\left( {\frac{k}{{k_{\max } }}} \right)^{2} + 2*(\omega_{\min } - \omega_{\max } )*\left( {\frac{k}{{k_{\max } }}} \right) + \omega_{\max } .$$

As the number of iterations increases, it can be seen from Fig. 7a that the optimization process eventually falls into the local optimum. The final fitting result of the parameters to be fitted is $[I_{Gi \to Th},g_{T},E_{T} ]$ = [− 3.4977, 3.0033, 129.6313], in which the final value of $E_{T}$ is far from the target value. In Fig. 7b, the optimization process never converges. When the upper limit of the iteration number is 50, the result is $[I_{Gi \to Th},g_{T},E_{T} ]$ = [− 3.5102, 6.2482, 114.1879]. The optimization process converges quickly in Fig. 7c, but falls into local optimum subsequently, and the final result is $[I_{Gi \to Th},g_{T},E_{T} ]$ = [− 3.4903, 4.6669, 113.8489]. It can be seen from Fig. 7d that in the particle swarm optimization algorithm, selecting a single nonlinear concave function as the inertia weight for parameter fitting is not necessarily better than selecting a single linear function. When the upper limit of the iteration number is reached, the fitting result is $[I_{Gi \to Th},g_{T},E_{T} ]$ = [− 3.5435, 5.8092, 112.7361].

**Figure 8:**
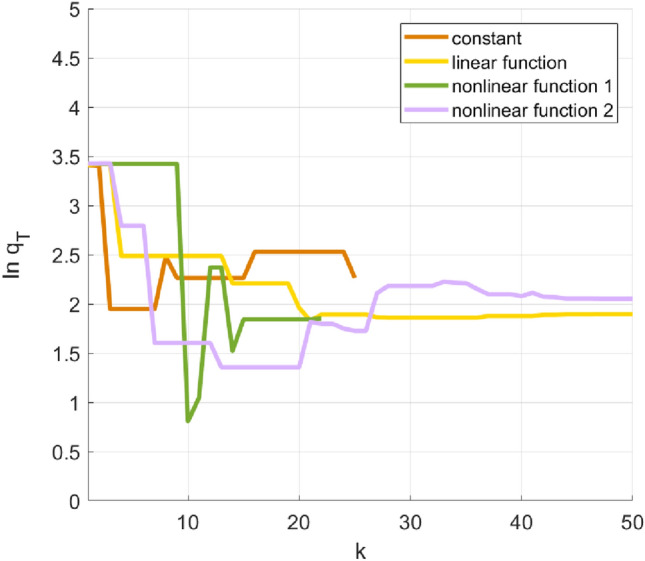
The fitness function changes with the iteration number when the inertia weight is a constant, a single determined linear function, and two sets of dynamic nonlinear concave functions separately.

When the inertia weight is selected as a constant, a single determined linear function and two sets of dynamic nonlinear concave functions, the fitness functions change with the number of iterations as shown in Fig. 8. It can be seen from the figure that as the number of iterations increases, the fitness function decreases faster when the inertia weight is a constant, but the final value is large; the fitness function decreases slowly when the inertia weight is a linear function; and the fitness function fluctuates down when the inertia weight is a nonlinear concave function. The fitness function converges faster when the first concave function is selected; the fitness function converges slowly when the second concave function is selected. When the iteration is terminated, the final fitness functions of the four cases are much larger than that of the improved particle swarm optimization algorithm proposed in this paper.

**Figure 9:**
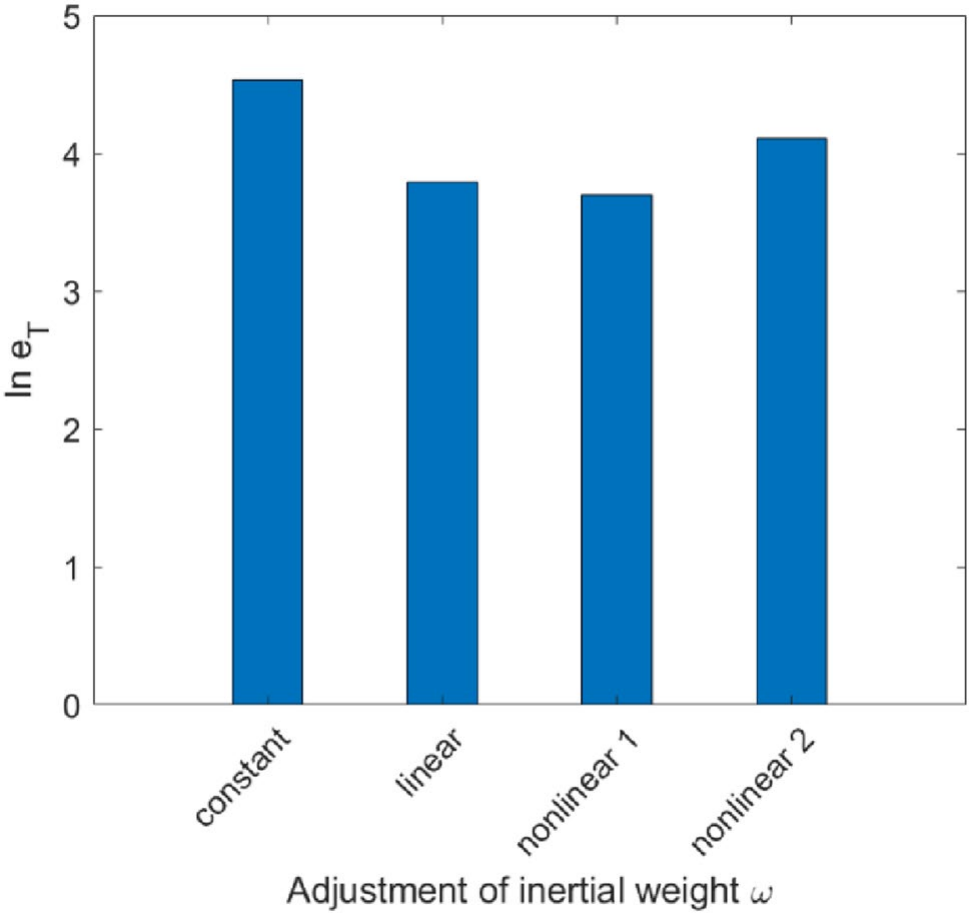
The parameter error comparison when the inertia weight is a constant, a single determined linear function, and two sets of dynamic nonlinear concave functions separately.

When the inertia weight is selected as a constant, a single determined linear function and two sets of dynamic nonlinear concave functions, the comparison chart of the total error of the three parameters $I_{Gi \to Th}$, $g_{T}$ and $E_{T}$ between the optimal solution and the given value is shown in Fig. 9. The natural logarithms of the total error in the four cases are [4.53, 3.79, 3.70, 4.11], which are much larger than that of the algorithm proposed in this paper.

It can be clearly seen from the comparisons that the method of combining dynamic nonlinear concave function and chaotic mapping adopted in this paper to construct inertial weight can avoid local optimum, and quickly converge to the optimal value, which can achieve good prediction characteristics.

The improved particle swarm optimization algorithm not only inherits the advantages of the particle swarm optimization algorithm, which has fast convergence speed and is easy to implement. It does not depend on the internal mechanism of the problem to be solved, which is very flexible in application. But it also avoids its disadvantages of tendency to fall into local optimum and appear premature convergence. The effectiveness of its search results only depends on the fitness function of the particles. So as long as the appropriate fitness function is selected, the data can be effectively fitted.

To conclude, a method of model parameter fitting based on neuron spiking characteristics is proposed in this paper. The traditional particle swarm optimization algorithm is improved, and the key parameters affecting neuron spiking characteristics are fitted using the improved particle swarm optimization algorithm. For thalamic neurons, three parameters that have a key impact on the Parkinson's state are fitted by using relay reliability as spiking characteristic index. Using the fitted parameters to reconstruct TC model, the spiking trajectories of the model can match well with the original model no matter in Parkinson's state or in normal state, which verifies the effectiveness of the fitting method. By comparing with the fitting effects of classical particle swarm optimization algorithm and several improved algorithms, it is shown that the proposed particle swarm optimization algorithm can better avoid local optimization and converge to the optimal value more quickly.

## Methods

### TC model and its key parameters

Signal relay related to Parkinson's disease is a very complex process. In order to study the dynamic mechanism of Parkinson's disease, researchers have established a conductance-based neuron model that simulates the relay process of thalamic neurons^25^. For the convenience of research, the TC model can be simplified to a single-compartment model^26^. The dynamic equation is as follows:

9 $$\begin{aligned} & C_{m} \frac{{dV_{Th} }}{dt} = - I_{L} - I_{Na} - I_{K} - I_{T} - I_{Gi \to Th} - I_{SM} \\ & I_{L} = g_{L} (V_{Th} - E_{L} ) \\ & I_{Na} = g_{Na} \cdot m_{\infty }^{3} (V_{Th} ) \cdot h_{Th} \cdot (V_{Th} - E_{Na} ) \\ & I_{K} = g_{K} \cdot 0.75(1 - h_{Th} )^{4} \cdot (V_{Th} - E_{K} ) \\ & I_{T} = g_{T} \cdot p_{\infty }^{2} (V_{Th} ) \cdot r_{Th} \cdot (V_{Th} - E_{T} ) \\ \end{aligned}$$

10 $$\begin{aligned} & \frac{{dh_{Th} }}{dt} = \frac{{h_{\infty } (V_{Th} ) - h_{Th} }}{{\tau_{h} (V_{Th} )}} \\ & h_{\infty } (V_{Th} ) = \frac{1}{{1 + \exp \left( {\frac{{V_{Th} + 41}}{4}} \right)}} \\ & \tau_{h} (V_{Th} ) = \frac{1}{{a_{h} (V_{Th} ) + b_{h} (V_{Th} )}} \\ & a_{h} (V_{Th} ) = 0.128\exp \left( { - \frac{{V_{Th} + 46}}{18}} \right) \\ & b_{h} (V_{Th} ) = \frac{4}{{1 + \exp \left( { - \frac{{V_{Th} + 23}}{5}} \right)}} \\ \end{aligned}$$

11 $$\begin{aligned} & m_{\infty } (V_{Th} ) = \frac{1}{{1 + \exp \left( { - \frac{{V_{Th} + 37}}{7}} \right)}} \\ & p_{\infty } (V_{Th} ) = \frac{1}{{1 + \exp \left( { - \frac{{V_{Th} + 60}}{6.2}} \right)}} \\ \end{aligned}$$

12 $$\begin{aligned} & \frac{{dr_{Th} }}{dt} = 2.5\frac{{r_{\infty } (V_{Th} ) - r_{Th} }}{{\tau_{r} (V_{Th} )}} \\ & r_{\infty } (V_{Th} ) = \frac{1}{{1 + \exp \left( {\frac{{V_{Th} + 84}}{4}} \right)}} \\ & \tau_{r} (V_{Th} ) = 28 + \exp \left( { - \frac{{V_{Th} + 25}}{10.5}} \right) \\ \end{aligned}$$

where $V_{Th}$ is the membrane potential of thalamic neurons. $C_{m}$ is the cell membrane capacitance. $I_{L}$, $I_{Na}$, $I_{K}$ and $I_{T}$ are leakage current, sodium ion current, potassium ion current and low-threshold calcium ion current, respectively. $I_{Gi \to Th}$ is the inhibitory current flowing from the basal ganglia of the brain to the thalamic neurons, and $I_{SM}$ is the excitatory input current of the thalamic neurons. $g_{L}$, $g_{Na}$, $g_{K}$, $g_{T}$ are the leakage conductance, the maximum conductance of sodium ion channel, the maximum conductance of potassium ion channel and the maximum conductance of low-threshold calcium ion channel, respectively. $E_{L}$, $E_{Na}$, $E_{K}$, $E_{T}$ are the corresponding reversal potenitals. $h_{Th}$ and $r_{Th}$ are the gating variables of the ion channels. $m_{\infty } (V_{Th} )$ and $p_{\infty }^{{}} (V_{Th} )$ are the steady state value of the sodium channel gating variable and low-threshold calcium channel gating variable. $\tau_{h} (V_{Th} )$ and $\tau_{r} (V_{Th} )$ are the time constants of the gating variables.

As the inhibitory current $I_{Gi \to Th}$ from the basal ganglia to the thalamus neurons increases, the excitatory relay signal of the cortex is weakened, leading to Parkinson's state. And the low-threshold calcium current $I_{T}$ also has an important impact on the relay function of thalamic neurons. So we select $I_{Gi \to Th}$, $g_{T}$ and $E_{T}$ as the key parameters of the model in this paper. The values of other parameters are detailed in literature ^26^. By fixing other parameter values, the TC model is abstracted into a model to be fitted with three variables.

### Improved particle swarm optimization algorithm

Particle swarm optimization algorithm is an intelligent optimization algorithm proposed by Kenney and Eberhart^22^. In order to avoid particles falling into local optimum during the search process, or appearing premature convergence, this paper adopts the method of combining nonlinear concave function and Logistic chaotic mapping to adjust the inertia weight of particles.

The fitness function of the particle is used as the criterion to judge the position of the particle. When the fitness function of a particle is smaller than the average fitness function of the particle population, that is, when the particle is evolving faster than other particles, we choose a certain nonlinear concave function to adjust the inertia weight of the particle to ensure that the convergence speed of the particle will not be too fast and lead to premature convergence. The concave function is selected because a large-scale global search is required at the beginning, so the function curve is relatively steep; later it needs to change from a large-scale global search to a small-scale fine search, so the function curve is relatively smooth. When the fitness function of the particle is larger than the average fitness function of the particle population, that is, when the evolution speed of the particle is slower than other particles, in order to avoid the particle from falling into the local optimum, we choose the non-deterministic Logistic chaotic mapping function^27^ to adjust the inertia weight to force the particle to jump out of the local optimum and continue to search for the global optimum.

The steps of the improved particle swarm optimization algorithm proposed in this paper are as follows:

I. Initialize the particle swarm;
II. Calculate the fitness function $q_{id}^{k}$ of each particle and the average fitness function $q_{avg}^{k}$ of the particle swarm;
III. If $q_{id}^{k} < q_{avg}^{k}$, meaning that the evolution speed of the particle is faster than other particles. We choose a nonlinear concave function to modify the inertia weight to make sure that the function curve is relatively steep at the beginning because large-scale global search is required, and relatively smooth at the end since it needs to change to a small-scale fine search:
  13 $$\omega = \omega_{\min } *\left( {\frac{{\omega_{\max } }}{{\omega_{\min } }}} \right)^{{\frac{1}{{1{ + }10*\left( {\frac{k}{{k_{\max } }}} \right)}}}}$$
  where $k$ is the current iteration number and $k_{\max }$ is the maximum iteration number. $\omega_{\max }$ and $\omega_{\min }$ are the maximum and minimum values of the inertia weight, respectively.
  If $q_{id}^{k} > q_{avg}^{k}$, meaning that the particle is evolving slower than other particles. We choose a non-deterministic Logistic chaotic mapping function to modify the inertia weight to make the particle to jump out of the local optimum:
  14 $$\begin{aligned} & \alpha = \alpha_{\max } - (\alpha_{\max } - \alpha_{\min } )\left( {\frac{k}{{k_{\max } }}} \right) \\ & \omega = \alpha + (1 - \alpha ) \cdot L_{map} \\ \end{aligned}$$
  where $\alpha$ is the adjustment factor of inertia weight which value range is [0,1], $\alpha_{\max }$ and $\alpha_{\min }$ are the maximum and minimum values of $\alpha$ respectively. $L_{map}$ is the result of Logistic chaotic mapping.
IV. Update the position and velocity of the particles according to the following formula:
  15 $$\begin{aligned} & v_{id} \left( {t + 1} \right) = \omega \times v_{id} (t) + c_{1} r_{1} \times (p_{id} (t) - x_{id} (t)) + c_{2} r_{2} \times (p_{gd} (t) - x_{id} (t)) \\ & x_{id} \left( {t + 1} \right) = x_{id} (t) + v_{id} (t + 1) \\ & if\;\left| {v_{id} } \right| > \left| {v_{\max } } \right|,\left| {v_{id} } \right| = \left| {v_{\max } } \right|. \\ \end{aligned}$$
  where $i = 1,2,...,N$, $d = 1,2,...,D$.${\text{x}}_{{{\text{id}}}}$, $v_{{{\text{id}}}}$ and ${\text{p}}_{{{\text{id}}}}$ respectively represent the position, velocity and historical optimal position of the ${\text{d}}$-dimensional search space of the ${\text{i}}$th particle, and ${\text{p}}_{{{\text{gd}}}}$ represents the historical optimal position of the entire particle population. $c_{1}$ and $c_{2}$ are constants, which are cognitive factor and social factor respectively, usually $c_{1} = c_{2} = 2$. $r_{1}$ and $r_{2}$ are uniformly distributed random numbers in the range of 0 to 1. $\omega$ is the inertia weight. $v_{{{\text{max}}}}$ is the upper limit of the particle speed, when the particle speed $\left| {v_{id} } \right| > \left| {{\text{v}}_{{{\text{max}}}} } \right|$, then $\left| {v_{id} } \right| = \left| {{\text{v}}_{{{\text{max}}}} } \right|$.
V. Determine whether the iteration termination condition is reached, that is, whether the maximum iteration number is reached or the fitness function of the particles is less than a certain threshold. If yes, the iteration ends; if not, repeat steps II to V until the iteration ends.

The ${\text{p}}_{{{\text{gd}}}}$ obtained in the last iteration is the optimal result to be searched.
